# Early RSV infection aggravates asthma-related Th2 responses by increasing the number of CD4 ^+^ TRM cells through upregulation of PLZF: Asthma aggravation induced by early RSV infection

**DOI:** 10.3724/abbs.2024220

**Published:** 2024-12-05

**Authors:** Meng Zhang, Jiafeng Sha, Na Li, Jingjing Feng, Tianyun Shi, Yunxia Yu, Xiaoting Ren, Zhoufang Mei, Zhijun Jie

**Affiliations:** 1 Department of Pulmonary and Critical Care Medicine Shanghai Fifth People’s Hospital Fudan University Shanghai 200240 China; 2 Department of Medicine Respiratory Emergency and Intensive Care Medicine The Affiliated Dushu Lake Hospital of Soochow University Suzhou 215128 China; 3 Center of Community-Based Health Research Fudan University Shanghai 200032 China

**Keywords:** asthma, respiratory syncytial virus infection, PLZF, tissue-resident memory T cell

## Abstract

Respiratory syncytial virus (RSV) infection is correlated with the chronic pathogenesis and exacerbation of asthma. However, the mechanism remains unclear. In this study, acute and memory (Mem) asthma models with early RSV infection are established to explore the persistence of the effects of RSV infection on asthma. Intravascular injection of an anti-CD45 antibody is performed to define CD4 ^+^ TRM cells accurately. RSV infection has a sustained impact on asthma exacerbation for at least six weeks, with high Th2 cytokine secretion in lung tissue instead of IgE response-related B cells. CD45 ^–^CD4 ^+^ TRM cells are positively correlated with RSV-related asthma exacerbation and severe airway inflammation. Mechanistically, overexpression of the transcription factor PLZF *in vitro* increases the number of CD4 ^+^ TRM cells, and conditional knockout of *Zbtb16* (encoding PLZF) can decrease the number of CD4 ^+^ TRM cells to aggravate allergic inflammation and reduce Th2 responses. This study provides evidence for potential combined strategies that might benefit asthma patients.

## Introduction

Respiratory syncytial virus (RSV) infection usually causes self-limited infections of the lung and respiratory tract. However, many studies have shown that children suffering from severe early RSV infections may have a higher incidence of recurrent wheezing and asthma in later childhood [1]. Children under 2 years of age with lower respiratory tract infections caused by RSV [2] are more susceptible to asthma [ 3, 4] . The mechanism is not entirely clear. RSV-induced bronchiolitis can damage airways, promote airway obstruction and recurrent wheezing, and increase the risk for subsequent asthma [5]. Th2-related inflammation is one of the key pathogenic factors of allergic asthma [6]. RSV infection in early infancy was also found to preferentially promote Th2-like responses in the nose, with local production of IL-4 and IL-5 [7]. Many experiments have indicated that RSV infection can exacerbate asthma through the Th2 immune response, but the duration of its effects and the underlying molecular mechanism have not been elucidated [ 8, 9] .

Several reports have suggested that CD4 ^+^CD44 ^+^ memory T cells are involved in RSV infection and asthma [ 10– 12] . There are several subtypes of memory T cells, including circulating memory T cells [i.e., effector memory T cells (TEMs) and central memory T cells (TCMs)] and tissue-resident memory T cells (TRMs) [ 13, 14] . TRMs reside permanently in peripheral tissues, such as the skin, lung, and gut mucosa, where they provide localized immune protection without circulation through the blood or lymphatic system [15]. TRMs are generated *in situ* and provide rapid and persistent protection by proliferating and producing effector molecules such as cytokines ( *e*. *g*., IFN-γ) and chemokines [16]. Conversely, TRMs have been shown to be key pathogenic factors in recurrent asthma in both BALB/c and C57 mice [ 16– 18] .

PLZF is a sequence-specific DNA-binding protein containing an N-terminal BTB/POZ domain and a C-terminal cluster of Krüppel-like C2H2 zinc fingers [19]. Interestingly, the functional state of PLZF can be altered in different cells and at different phases. PLZF expression at the innate lymphoid cell precursor stage promotes the functional properties of mature ILC2 proliferation for the innate allergic response [20]. Our previous study revealed that PLZF facilitates iNKT cell recruitment to the lung to promote immune tolerance to prevent asthma [21]. On the other hand, PLZF is essential for CD4 ^+^ T-cell development and can regulate the memory phenotype of CD44 ^+^ CD4 ^+^ T cells to promote the development of asthma tolerance [22]. These observations suggest that PLZF in CD4 ^+^ T cells influence the development of memory T cells and their expression, consequently affecting immune tolerance.

To date, researchers have not clearly determined whether TRMs are involved in asthma exacerbation induced by early RSV infection. The mechanism of CD4 ^+^ TRM cell development has not yet been fully characterized. This study aimed to investigate the role of CD4 ^+^ TRMs in immune tolerance via asthma models. The results showed that the proportion of CD4 ^+^CD44 ^+^ T cells, especially CD4 ^+^ TRMs, is positively correlated with the severity of asthma symptoms caused by RSV infection, as well as several other molecular markers. With increasing resting time, the persistent influence of RSV infection on the pathogenesis of asthma is closely related to CD4 ^+^ TRMs. The transcription factor PLZF could facilitate the development of CD4 ^+^ TRMs, providing a novel and promising therapeutic target for asthma.

## Materials and Methods

### Animals

Three- to four-week-old female BALB/c and C57 mice were purchased from East China Normal University Animal Center (Shanghai, China). Zbtb16-flox mice (strain no. T016043) were purchased from GemPharmatech (Nanjing, China). CD4-Cre mice (strain no. 022071) were purchased from the Jackson Laboratory (Bar Harbor, USA). The mice were raised in groups at 21-24°C with 12/12-h dark/light cycles and with free access to food and water under specific-pathogen-free conditions. The mice were randomly assigned to each group and allowed to acclimate substantially for 1 week before the experiments. Anaesthesia was induced via an intraperitoneal (i.p.) injection of pentobarbital sodium (50 mg/kg) before the mice were sacrificed. All the animal experiments were approved by the Animal Welfare and Ethics Group, Department of Laboratory Animal Science, Fudan University (Animal Ethics Review Number: 2020 Shanghai Fifth People’s Hospital JS-031).

### Establishment of asthma and RSV-infected asthma mouse models

**Figure 1:**
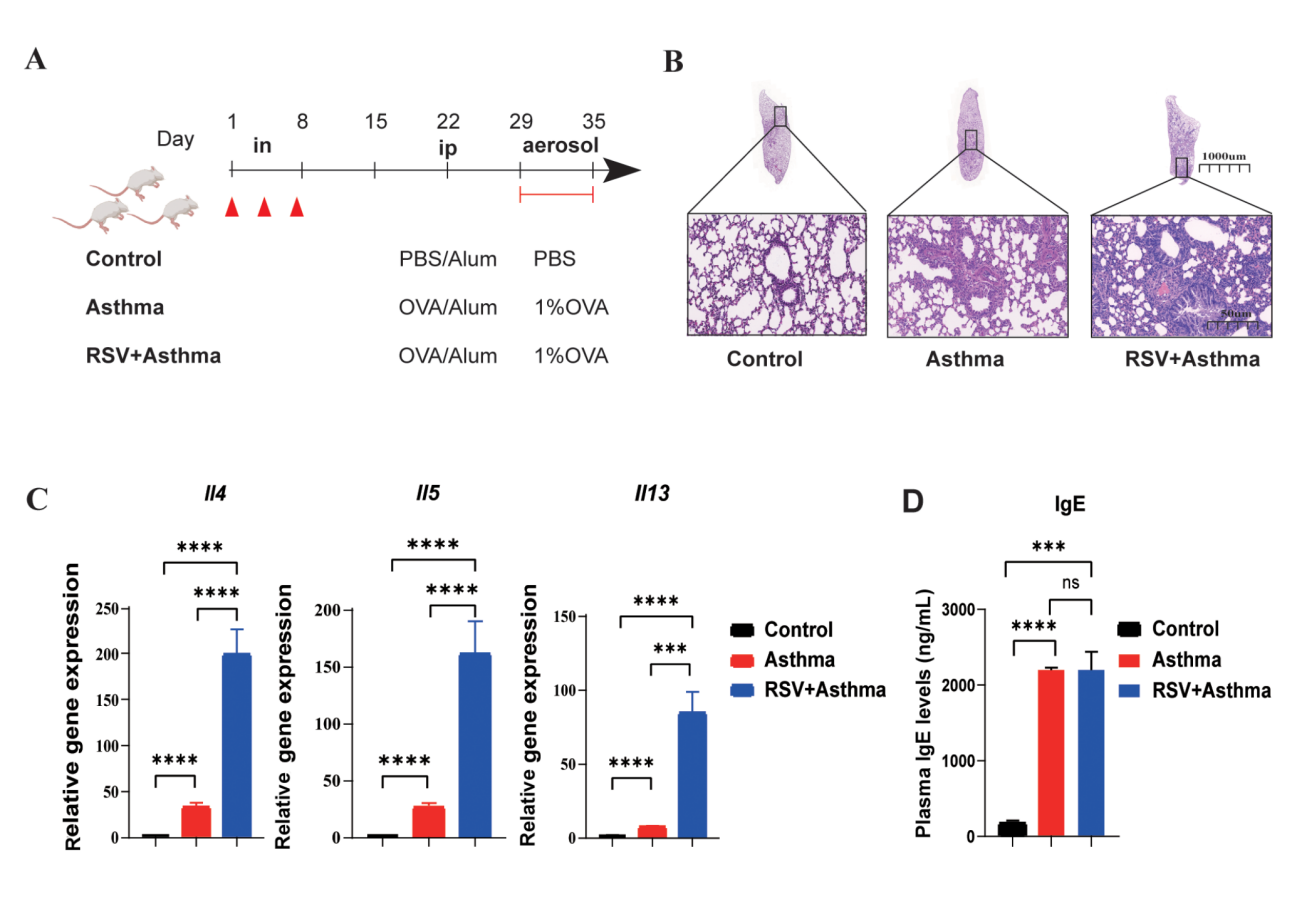
Figure 1 Early RSV infection aggravates allergic lung inflammation and elevates Th2 cytokine levels (A) Establishment of the RSV + asthma and asthma model. (B) Representative microphotographs of lung histological sections from control, RSV + asthma and asthma mice at objective magnifications of 1× and 29×. (C) The gene expression levels of Il4,Il5 and Il13 in the whole lung were measured by real-time qPCR. (D) Plasma IgE levels were detected by ELISA. Data are shown as the mean ± SEM. *P < 0.05, **P < 0.01, ***P < 0.001, ****P < 0.0001, ns: not significant (unpaired two-tailed Student’s t test). The results are from one representative experiment of at least three independent experiments in (C,D) (n = 3–6 in each group).

**Figure 2:**
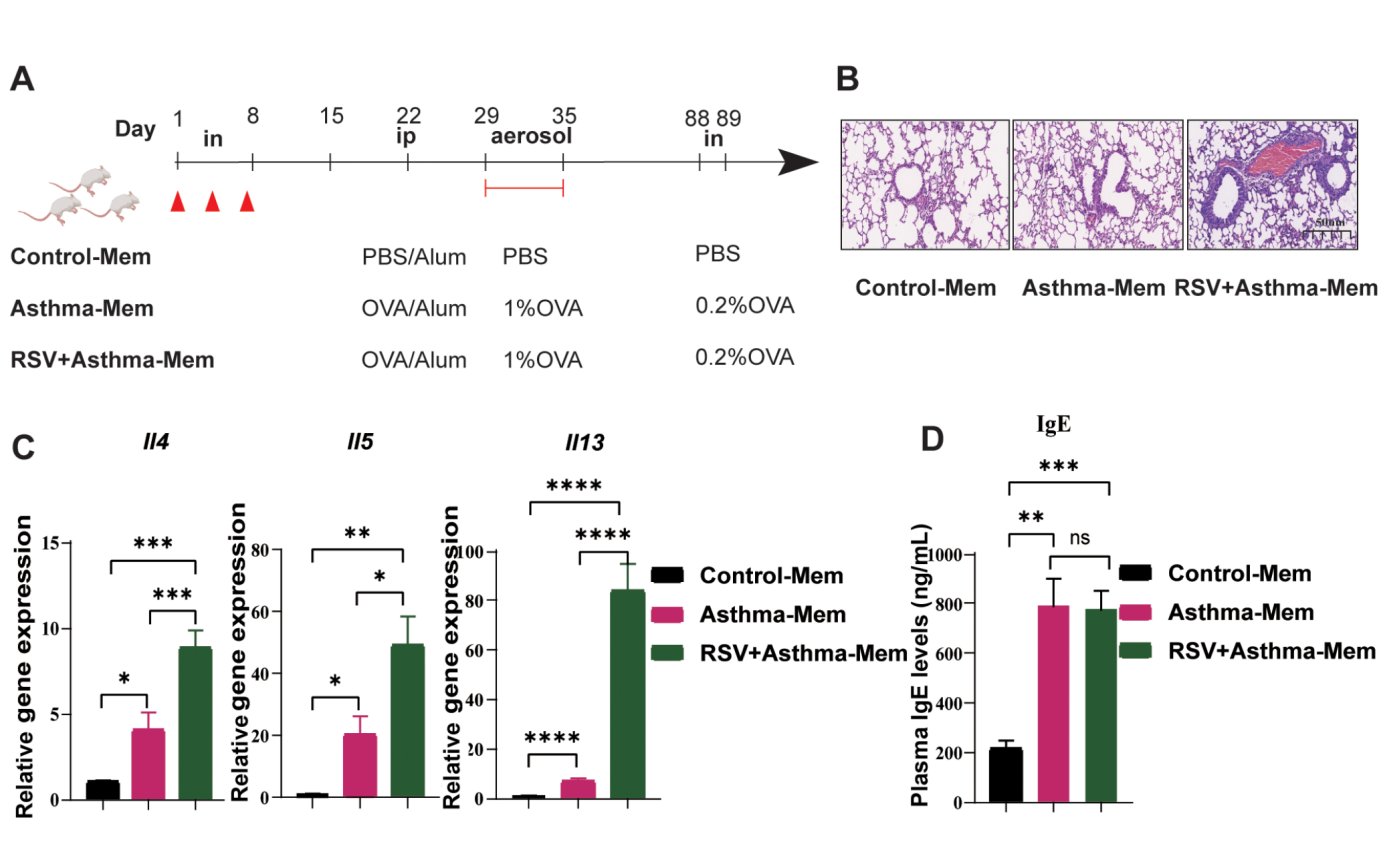
Figure 2 The aggravation of asthma caused by early RSV infection is maintained for at least six weeks (A) Establishment of the RSV-infected asthma-Mem and asthma-Mem models. (B) Representative microphotographs of histological lung sections from control-Mem, RSV-infected asthma-Mem and asthma-Mem mice at objective magnifications of 1× and 29×. (C) Gene expression levels of Il4,Il5 and Il13 in the whole lung were measured by real-time qPCR. (D) Plasma IgE levels were detected by ELISA. Data are shown as the mean ± SEM. *P < 0.05, **P < 0.01, ***P < 0.001, ****P < 0.0001, ns: not significant (unpaired two-tailed Student’s t test). The results are from one representative experiment of at least three independent experiments in (C,D) (n = 3–6 in each group).

The BALB/c mice (3–4 weeks old) were divided into three groups (Figures 1 A and 2 A). (i) Control: after 2 weeks, one dose of alum (50 μL of alum gel in 70 μL of PBS) was intraperitoneally administered to the mice on the 22nd day, and the mice were finally aerosolized with PBS (40 min/d) from days 29 to 35. (ii) Asthma: After 2 weeks, one dose of OVA/alum (50 μg of OVA and 50 μL of alum gel in 70 μL of PBS) was intraperitoneally administered to the mice on the 22nd day, and the other dose of alum was aerosolized with OVA (10 mg/mL in PBS, 40 min/d) from days 28 to 35. (iii) RSV + asthma: on days 1, 4, and 7, before the establishment of asthma, 25 μL of RSV was dripped into each nostril twice (giving a total of 100 μL per mouse), after which the mice were allowed to rest for 2 weeks, and then one dose of OVA/alum was intraperitoneally administered to the mice on the 22nd day, and aerosolized with OVA (10 mg/mL in PBS, 40 min/d)

Six weeks of rest were given to these three groups of mice, and then at days 88 and 89, 50 μL of 0.2% OVA was intranasally added to the asthma and RSV + asthma groups of mice, which were referred to as asthma-memory (asthma-Mem) and RSV + asthma-memory (RSV + asthma-Mem) groups. The mice were sacrificed on day 90.

### RSV titration and infection

Hep2 cells (ATCC, Rockville, USA) were cultivated in Dulbecco’s modified Eagle’s medium (DMEM) supplemented with 10% (v/v) fetal bovine serum. The A2 strain of RSV was kindly supplied by the School of Basic Medical Sciences of Fudan University (Shanghai, China). When the cell confluence reached approximately 80%, RSV stored in liquid nitrogen was used to infect the Hep2 cells. When obvious lesions were observed (approximately 4–5 days later and nearly all the cells at the bottom of the culture flask had died), RSV was collected, and the virus titre was determined via a standard plaque assay on Hep2 cells. The RSV was subpackaged and placed in a –80°C freezer for later use.

On days 1, 4, and 7 before the establishment of asthma, 25 μL of RSV was dripped into each nostril twice (for a total of 100 μL per mouse). During the nasal drip, the mice were prevented from suffocating. Asthma and RSV-infected asthma mouse models were then compared.

### Collection of BALF

BALF was collected via a tracheal incision via a process that involved washing the lungs three times with 1 mL of PBS. After centrifugation at 500 *g* for 15 min at 4°C, BALF cells were collected from the sediment for counting, and the supernatant was stored at –80°C for subsequent examination of eosinophilic granulocytes.

### Real-time qPCR analysis

Total RNA was extracted from the lungs via the AFTSpin Tissue/Cell Fast RNA Extraction kit for animals (ABclonal, Wuhan, China) following the manufacturer’s instructions. The RNA concentration was determined using a NanoDrop 2000 spectrophotometer (Thermo Fisher Scientific, Waltham, USA). cDNA was synthesized with ABScript III RT Master Mix for quantitative polymerase chain reaction (qPCR) with a gDNA Remover kit (ABclonal) and then subjected to RT-qPCR analysis with Taq Pro Universal SYBR qPCR Master Mix (Q712-02; Vazyme, Nanjing, China). The sequences of the primers used are shown in Table 1.

**Table:** **Table 1** The sequences of the primers used in this study

Gene	Primer sequence (5′→3′)
*Il4*	Forward: ATCATCGGCATTTTGAACGAGGTC
	Reverse: ACCTTGGAAGCCCTACAGACGA
*Il5*	Forward: ATCATCGGCATTTTGAACGAGGTC
	Reverse: ACCTTGGAAGCCCTACAGACGA
*Gapdh*	Forward: CATCACTGCCACCCAGAAGACTG
	Reverse: ATGCCAGTGAGCTTCCCGTTCAG
*Il13*	Forward: AAAGCAACTGTTTCGCCACG
	Reverse: CCTCTCCCCAGCAAAGTCTG
*Zbtb16*	Forward: CTGGGACTTTGTGCGATGTG
	Reverse: CGGTGGAAGAGGATCTCAAACA
*Il15*	Forward: GTAGGTCTCCCTAAAACAGAGGC
	Reverse: TCCAGGAGAAAGCAGTTCATTGC
*Gata3*	Forward: CCTCTGGAGGAGGAACGCTAAT
	Reverse: GTTTCGGGTCTGGATGCCTTCT
*Muc5ac*	Forward: CCACTGGTTCTATGGCAACACC
	Reverse: GCCGAAGTCCAGGCTGTGCG
*Bcl6*	Forward: CAGAGATGTGCCTCCATACTGC
	Reverse: CTCCTCAGAGAAACGGCAGTCA

### Single-cell suspensions

The mice were intravascularly injected with 3 mg of APC/Cyanine7-conjugated anti-mouse CD45 (BioLegend, San Diego, USA) in 300 μL of Dulbecco’s phosphate-buffered saline (DPBS) [23]. After 10–15 min, the mice were sacrificed. The entire lungs were digested with 50 μg/mL Liberase (Roche, Basel, Switzerland) and 1 μg/mL DNase I at 37°C for 50 min with shaking (200 rpm). The digested lung tissue was subsequently centrifuged at 500 *g* for 7 min at 4°C. After the erythrocytes were lysed for 7 min, the cells were collected with red blood cell lysis buffer (Beyotime, Shanghai, China). Finally, a single-cell suspension was obtained with a 70 μM cell strainer (BD Biosciences, Franklin Lakes, USA). Similarly, the spleen was ground with a 70 μM cell strainer (BD Biosciences) and then centrifuged at 500 *g* for 7 min at 4°C. Later, red blood cell lysis buffer (Beyotime) was used to lyse red blood cells for 10 min. Finally, a single-cell suspension was obtained with a 40 μM cell strainer (BD Biosciences).

### Flow cytometry

The cell suspension was incubated with purified rat anti-mouse CD16/CD32 (Clone 2.4G2; BD Biosciences) to block Fc fragments on immune cells for 15 min at 4°C. Before surface staining, the cells were stained with a Zombie UV™ Fixable Viability kit (BioLegend) in PBS (1:100) for 20–30 min at room temperature in the dark to distinguish live cells. Next, monoclonal antibodies against surface antigens were utilized to detect the surface markers of the cells through incubation with the cell suspension for 30 min at 4°C in the dark. The key materials including Brilliant Violet 510™ anti-mouse CD3ε (17A2), FITC anti-mouse CD4 antibody (RM4-5), PerCP/Cyanine5.5 anti-mouse/human CD44 (IM7), APC anti-mouse CD62L (MEL-14), PE/Cyanine7 anti-mouse CD69 (H1.2F3), and Compensation Beads were all from BioLegend.

A transcription factor buffer set (BD Biosciences) was used for intranuclear staining. The nuclear membranes were fixed and permeabilized with 1× fix/perm buffer for 40–50 min at 4°C. PE-conjugated anti-mouse PLZF antibody (9E12; BioLegend) was used to suspend the cells in 100 μL of 1× perm/wash buffer at 2–8°C for 40–50 min in the dark.

All samples were filtered through a 40-μm cell strainer prior to flow cytometry experiment. Data were acquired with an LSR Fortessa™ flow cytometer (BD Biosciences) and Beckman Coulter CytoFlex (Beckman Coulter, Pasadena, USA). All the data were analyzed with FlowJo software (TreeStar, Ashland, USA) and CytExpert.

### Collection of plasma and IgE detection

The mice were anesthetized, and approximately 0.5–1 mL of cardiac blood was obtained via the use of ethylenediaminetetraacetic acid (EDTA) to prevent coagulation. After centrifugation at 1500 *g* for 10 min at 4°C, the upper layer (plasma) was transferred into a 1.5-mL tube and stored in a –80°C freezer. The IgE levels in the plasma were detected using a mouse IgE enzyme-linked immunosorbent assay (ELISA) set (BD OptEIA™; BD Biosciences) following the supplier’s instructions.

### Isolation and culture of lymphocytes from the spleen, plasmid transfection and treatment procedures

Eight-week-old wild-type C57BL/6J mice were euthanized, and the spleens of each mouse were removed and ground gently on a 70-μm cell strainer with 5–6 mL of mouse lymphocyte separation medium (Dakewe, Shenzhen, China) in a 15-mL centrifuge tube. The mixture was used for density gradient separation of spleen lymphocytes. The lymphocytes were counted with a hemocytometer and cultured in RPMI 1640 containing 10% (v/v) FBS *in vitro*. Approximately 24 h later, the lymphocytes were transfected with empty DNA plasmids or DNA plasmids expressing Zbtb16 through Polyplus-DNA-transfection® reagent (Polyplus, Orléans, France) for 24–72 h. After 48 h, flow cytometry and RT-qPCR were performed to test the transfection and *Zbtb16*-knockdown efficiency. After verification, at 48 h, the supernatant of OVA-treated BEAS-2B cells (for 36 h, BEAS-2B cells were normal bronchial epithelial cells that were suitable for OVA stimulation *in vitro*) was prepared, and untreated BEAS-2B cells were utilized to stimulate Zbtb16-overexpressing lymphocytes and NC-controls for 12 h. Thereafter, the cells were collected and then stained with antibodies for flow cytometric analysis.

### Statistical analysis

All the data were analyzed using Prism 8.0 software (GraphPad Software, San Diego, USA). Data are expressed as the mean±standard error of the mean (SEM). Differences between two groups were analyzed using an unpaired two-tailed Student’s *t* test. *P*<0.05 was considered to indicate statistical significance.

## Results

### Early RSV infection strongly exacerbates asthma for a long period of time, resulting in high Th2 cytokine secretion in the lungs

We first established pathological models (labelled asthma and RSV +  asthma) ( Figure 1A). RSV was administered on days 7, 4, and 1, followed by a two-week rest period to simulate early RSV infection in mice. Ovalbumin [24] is used as an allergen to stimulate the immune response and induce pulmonary allergic inflammation ( Figure 1A). Classic phenotypes of allergic asthma, including inflammatory cell infiltration and smooth muscle contraction, were observed in the airways of the asthma group after aerosol inhalation of OVA for seven days ( Figure 1B) [25]. Notably, the lungs of the RSV + asthma group exhibited more severe asthmatic symptoms, including swollen airway and inflammatory cell infiltration ( Figure 1B). We further examined the expressions of Th2 cytokines, including *Il4*, *Il5*, *and Il13*. Significant and dramatic elevation of these markers were detected only in the asthma and RSV+asthma groups, with the RSV + asthma group showing higher levels of Th2 cytokines than the asthma group did ( Figure 1C). Additionally, although the level of IgE secretion in asthmatic mice was significantly higher than that in control mice, there were no significant differences between asthmatic and RSV-infected asthmatic mice ( *P*  > 0.05; Figure 1D). All the markers mentioned above serve as indicators of the allergic response. In this study, we successfully constructed an allergic asthma model and confirmed the histologic and molecular features of exacerbated asthma symptoms due to early RSV infection. These findings suggested that the influence of RSV infection on asthma may rely more on Th2-related inflammation, but whether this influence is persistent remains unclear.

To further explore the lasting effects of early RSV infection on asthmatic symptoms, we extended the rest period to six weeks for both the asthma group and the RSV-infected asthma group. On Days 88 and 89, a low concentration of OVA was intranasally administered to the mice (labelled Mem) ( Figure 2A). Histological analysis revealed that the lungs of RSV-infected asthma-Mem mice remained in a pathological state, with more severe symptoms than those of asthma-Mem mice, which were slightly more severe than those of control-Mem mice ( Figure 2B). Similarly, compared with those of asthma-Mem mice, the lungs of RSV-infected asthma-Mem mice persistently secreted significantly higher levels of Th2 cytokines such as *Il4* and *Il5*, with no significant increase in IgE ( Figure 2C,D). We propose that early RSV infection has a lasting pathogenic influence on asthmatic lung tissue, which may be related to sustained high Th2 inflammation.

### High CD4 ^+^CD44 ^+^ memory T cells are positively correlated with persistent asthma exacerbation

**Figure 3:**
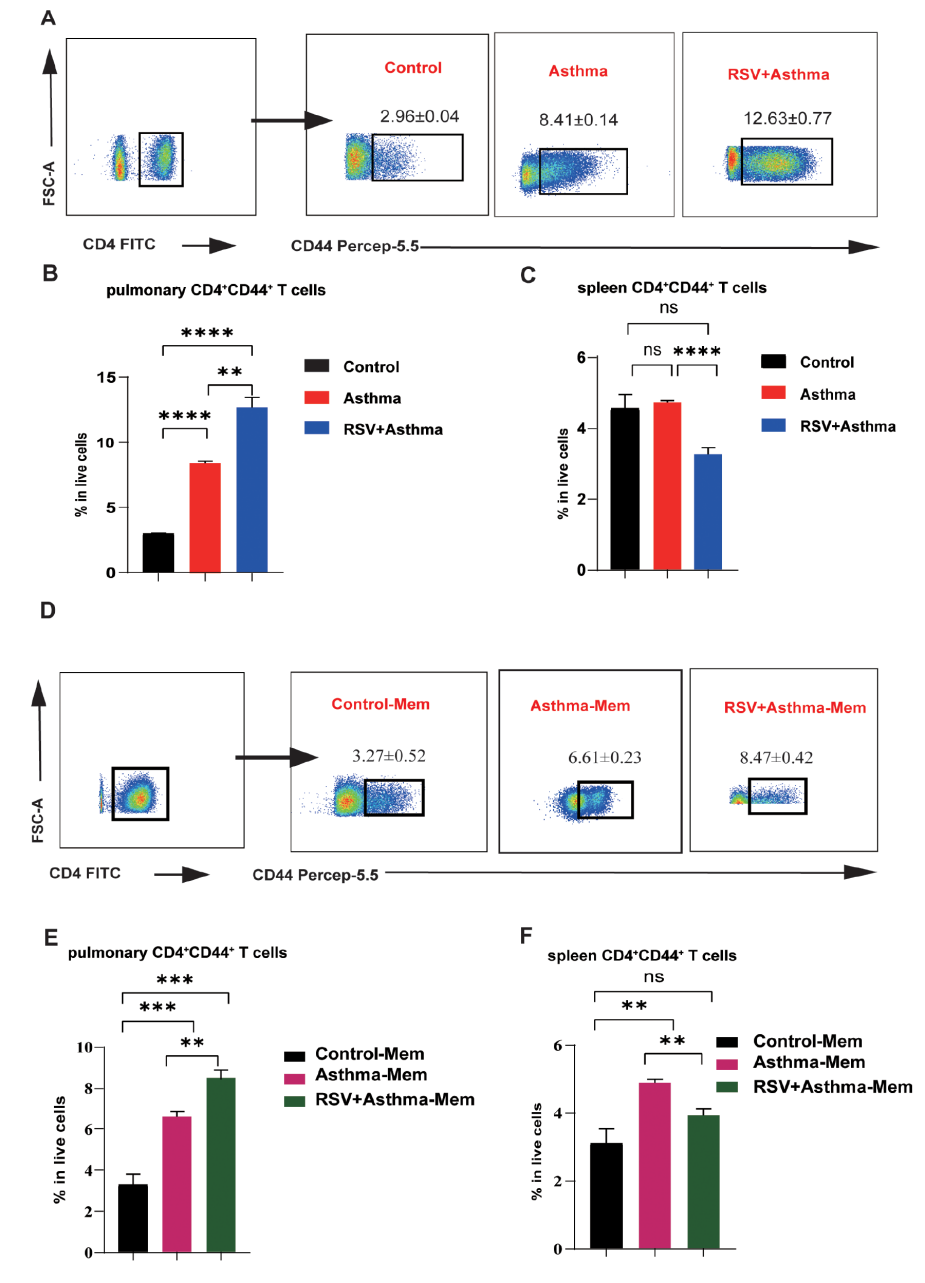
Figure 3 CD4 ^+^CD44 ^+^ T cells are greater in the RSV-infected asthma group than in the asthma group (A) Flow cytometric plots of CD4+CD44+ T cells gated from live CD4+ T cells in the lungs of control, asthma, and RSV + asthmatic mice. (B,C) Comparison of the proportions of CD4+CD44+ T cells among live cells in the lungs and spleens of control, asthma, and RSV + asthma mice detected by flow cytometry. (D) Flow cytometric plots of CD4+CD44+ T cells gated from live CD4+ T cells in the lungs of control-Mem, asthma-Mem, and RSV + asthma-Mem mice. (E,F) Comparison of the proportions of CD4+CD44+ T cells among live cells in the lungs and spleens of control-Mem, asthma-Mem, and RSV+asthma-Mem mice detected by flow cytometry. Data are shown as the mean ± SEM. *P < 0.05, **P < 0.01, ***P < 0.001, ****P < 0.0001, ns: not significant (unpaired two-tailed Student’s t test). The results are from one representative experiment of at least three independent experiments in(B,C,E,F) (n = 3–5 in each group).

To investigate the relationship between CD4 ^+^CD44 ^+^ memory T cells and allergic asthma, we performed flow cytometry analysis on three different models. Compared with those in asthmatic mice, CD4 ^+^CD44 ^+^ memory T cells were significantly enriched in the lungs of RSV-infected asthmatic mice, and both groups presented much greater numbers of CD44 ^+^ memory T cells than did the control mice ( Figure 3A,B). Re-stimulation was performed by intranasal delivery of a low concentration of OVA to determine whether the changes in CD4 ^+^CD44 ^+^ T cells are still correlated with the pathological state ( Figure 3D,E). Similarly, accompanied by severe allergic phenotypes, the proportion of CD4 ^+^CD44 ^+^ memory T cells increased again in RSV-infected asthma-Mem mice (Figures 2 B and 3 D,E). These differences were not detected in the spleen, which serves as the germinal zone of the circulating memory T cells, suggesting that a specific immune response occurred in the lung tissue of the acute and memory asthma and asthma groups with early RSV infection ( Figure 3C,F). Taken together, these findings suggest that the number of CD4 ^+^CD44 ^+^ memory T cells in lung tissue is highly positively correlated with the severe allergic symptoms of asthma induced by RSV infection, indicating a pulmonary-specific immune response.

### CD4 ^+^ TRMs account for the major change in CD4 ^+^CD44 ^+^ memory T cells

**Figure 4:**
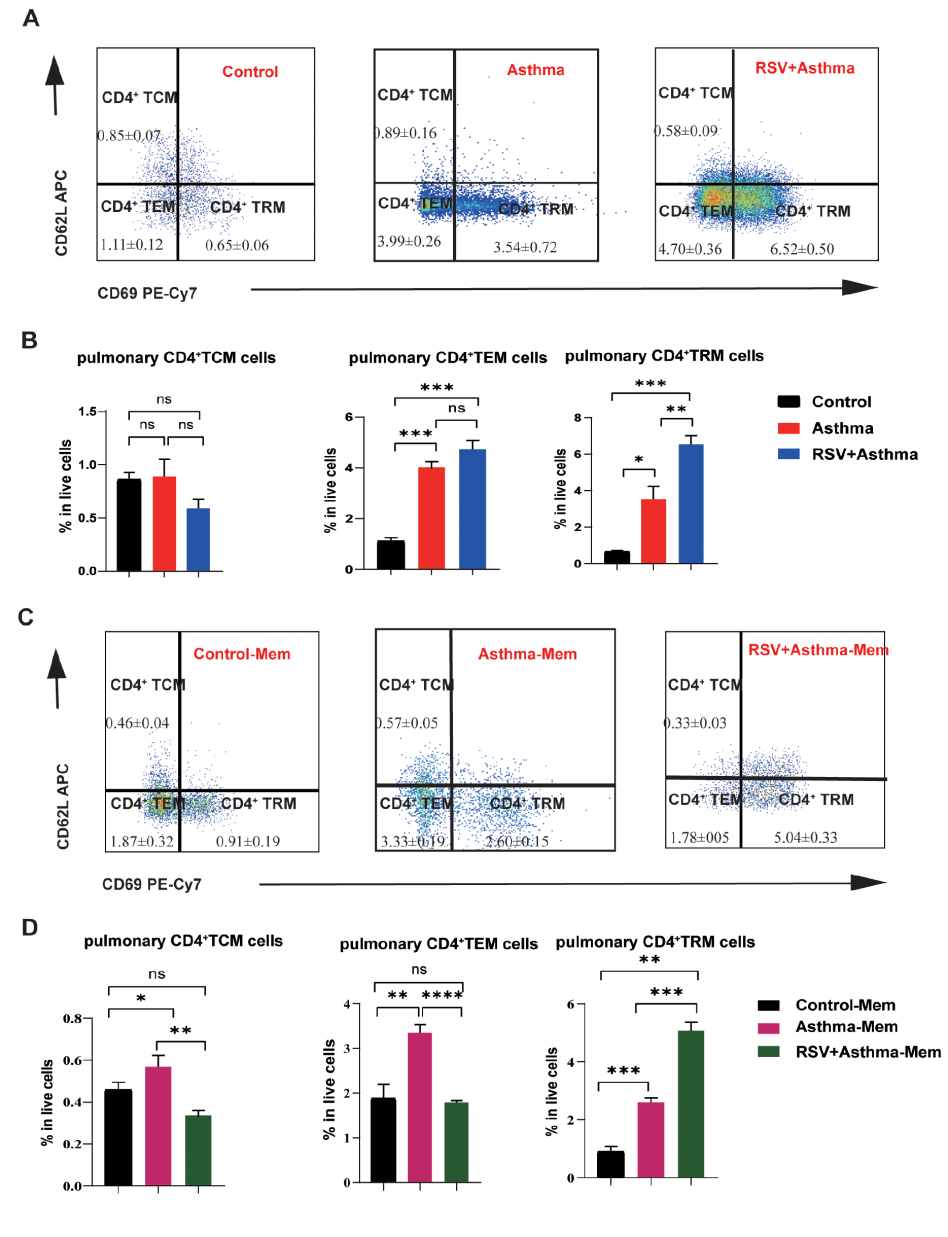
Figure 4 Early RSV-infected asthma induces persistently high expression of CD4+ TRMs (A) Flow cytometric plots of CD4+ TCMs (CD62L+CD69–), CD4+ TEM (CD62L–CD69–) cells and CD4+ TRM (CD62L+CD69+) cells gated from live CD4+CD44+ T cells in the lungs of control, asthma, and RSV + asthma mice. (B) Comparison of CD4+ TCMs, CD4+ TEMs and CD4+ TRMs in the lungs of control, asthma, and RSV+asthmatic mice. (C) Flow cytometric plots of CD4+ TCMs (CD62L+CD69–), CD4+ TEM (CD62L–CD69–) cells and CD4+ TRM (CD62L+CD69+) cells gated from live CD4+CD44+ T cells in the lungs of control-Mem, asthma-Mem, and RSV + asthma-Mem mice. (D) Comparison of CD4+ TCMs, CD4+ TEMs and CD4+ TRMs in the lungs of control-Mem, asthma-Mem, and RSV + asthma-Mem mice. Data are shown as the mean ± SEM. *P < 0.05, **P < 0.01, ***P < 0.001, ****P < 0.0001, ns: not significant (unpaired two-tailed Student’s t test). The results are from one representative experiment of at least three independent experiments in (B,D) (n = 3-6 in each group).

The homing molecules CD62L and CD69 were used for differentiation of different subsets of memory T cells [ 26– 28] . On the basis of the expressions of CD62L and CD69, CD4 ^+^CD44 ^+^ memory T cells can be categorized into three types: CD62L ^–^CD69 ^–^ TEMs, CD62L ^+^CD69 ^–^ TCMs, and CD62L ^–^CD69 ^+^ TRMs. It remains unclear which subtype is responsible for the changes in CD4 ^+^CD44 ^+^ memory T cells. After the allergic response was induced, the number of CD4 ^+^ TRMs was significantly greater in the RSV-infected asthma group than in the other groups ( Figure 4A,B). Similar results were obtained in the re-stimulation experiments ( Figure 4C,D).

**Figure 5:**
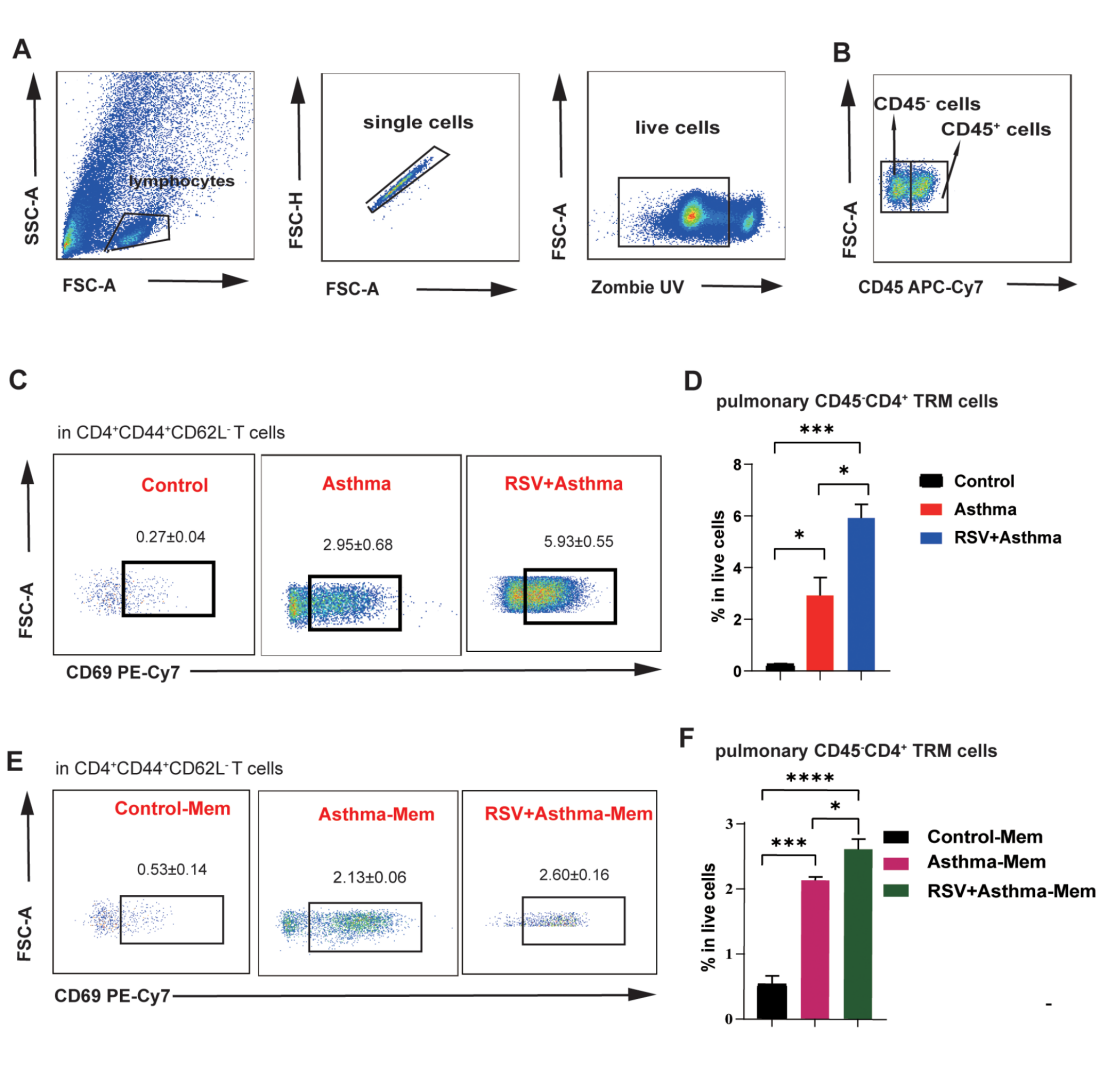
Figure 5 CD45 ^-^CD4 ^+^ TRMs dominantly contributes to the upregulation of CD4 ^+^CD44 ^+^ memory T cells (A,B) General panel logic of CD45+ and CD45– cells detected by flow cytometry. (C,D) Comparison of CD45–CD4+ TRMs in the lungs of control, asthma, and RSV+asthma mice. (E,F) Comparison of CD45–CD4+ TRMs in the lungs of control-Mem, asthma-Mem, and RSV + asthma-Mem mice. Data are shown as the mean ± SEM. *P < 0.05, **P < 0.01, ***P < 0.001, ****P < 0.0001, ns: not significant (unpaired two-tailed Student’s t test). The results are from one representative experiment of at least three independent experiments in (D,F) (n = 3–6 in each group).

Furthermore, to define CD4 ^+^ TRMs with limited cell surface markers sufficiently and precisely, we utilized the fluorescent antibody APC-Cy7 CD45 to discriminate vascular and tissue memory T cells through intravascular injection ( Figure 5A). CD45 is a critical cell surface marker expressed by leukocytes, and intravascular staining effectively discriminates between tissue-localized and blood-borne cells in non-lymphoid tissues [ 23, 29] . Significant enrichment of CD45 ^–^CD4 ^+^ TRMs was found in the lung tissue of RSV-infected asthmatic and asthma-Mem mice, suggesting the enrichment of tissue-localized cells ( Figure 5C–F). These results suggested that TRMs are responsible for the changes in CD4 ^+^CD44 ^+^ memory T cells in the pathogenesis of asthma exacerbation.

### PLZF promotes the development of CD4 ^+^ TRMs to prevent RSV-induced asthma aggravation

**Figure 6:**
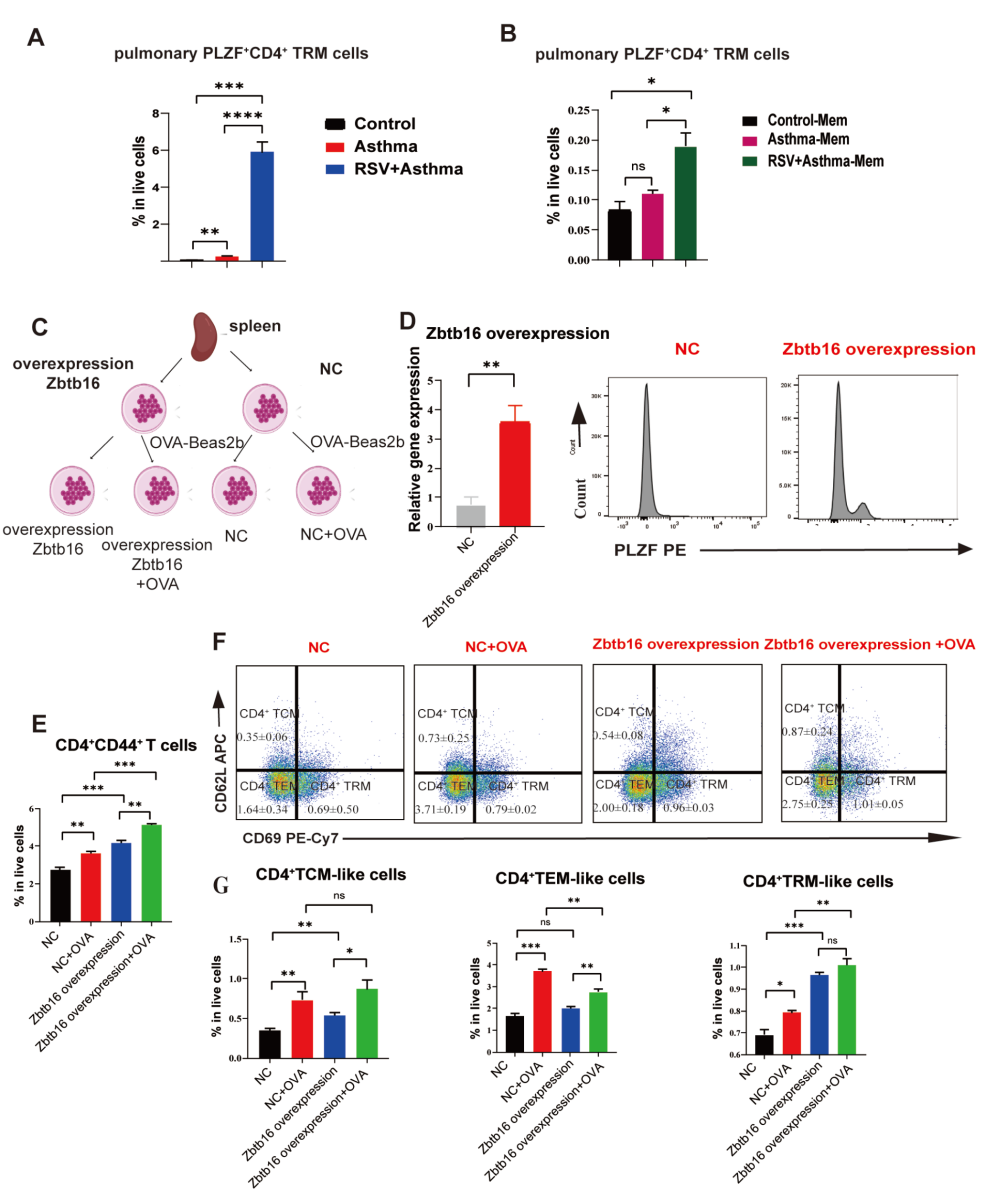
Figure 6 Overexpression of Zbtb16 *in vitro* increases the number of CD4 ^+^ TRMs (A) Comparison of pulmonary PLZF+CD4+ TRMs in the control, asthma and RSV-infected asthma groups. (B) Comparison of pulmonary PLZF+CD4+ TRMs in the lungs of Control-Mem, asthma-Mem and RSV-infected asthma-Mem mice. (C) Workflow of Zbtb16 overexpression and OVA-Beas2b stimulation of the splenic lymphocytes of BALB/c mice. (D) Validation of the overexpression of Zbtb16 (encoding PLZF) via real-time qPCR and flow cytometry. (E) Changes in CD4+CD44+ T cells among live cells stimulated with OVA-Beas2b. (F,G) Comparison of CD4+ TCM-like, CD4+ TEM-like and CD4+ TRM-like cells among live cells stimulated with OVA-Beas2b. Data are shown as the mean ± SEM. *P < 0.05, **P < 0.01, ***P < 0.001, ****P < 0.0001, ns: not significant (unpaired two-tailed Student’s t test). The results are from one representative experiment of at least three independent experiments in (A,B,D,E–G) (n = 3–6 in each group).

As mentioned above, PLZF is a critical transcription factor in the pathogenesis of asthma. PLZF can affect immune tolerance in asthma by regulating immune memory phenotypes, including CD4 ^+^ TEMs and CD4 ^+^ TCMs. Considering that memory T cells can be classified into three types, namely, CD4 ^+^ TCM, CD4 ^+^ TEM, and CD4 ^+^ TRMs, inspecting the regulatory role of PLZF in the development of CD4 ^+^ TRMs is highly important. As shown in Figure 6A,B, the number of pulmonary PLZF ^+^CD4 ^+^ TRMs in RSV-infected asthmatic mice was much greater than that in asthmatic mice, suggesting that PLZF likely regulated CD4 ^+^ TRMs in asthma exacerbation.

We collected and cultured spleen lymphocytes from wild-type (WT) mice (labelled NC) ( Figure 6C) and overexpressed PLZF through plasmid construction and transient transfection technology. The efficiency was verified by real-time qPCR and flow cytometry ( Figure 6D). Compared with that in the NC group, PLZF overexpression increased the CD4 ^+^CD44 ^+^ T-cell ratio in both the OVA-treated and non-treated groups ( Figure 6E). Among the CD4 ^+^ memory T cells, more CD4 ^+^ TRM-like cells were generated, suggesting that overexpression of PLZF significantly enhanced the development of CD4 ^+^ TRM-like cells *in vitro* ( Figure 6F,G). These results suggest that PLZF plays an upregulatory role in the development of CD4 ^+^ TRM-like cells.

**Figure 7:**
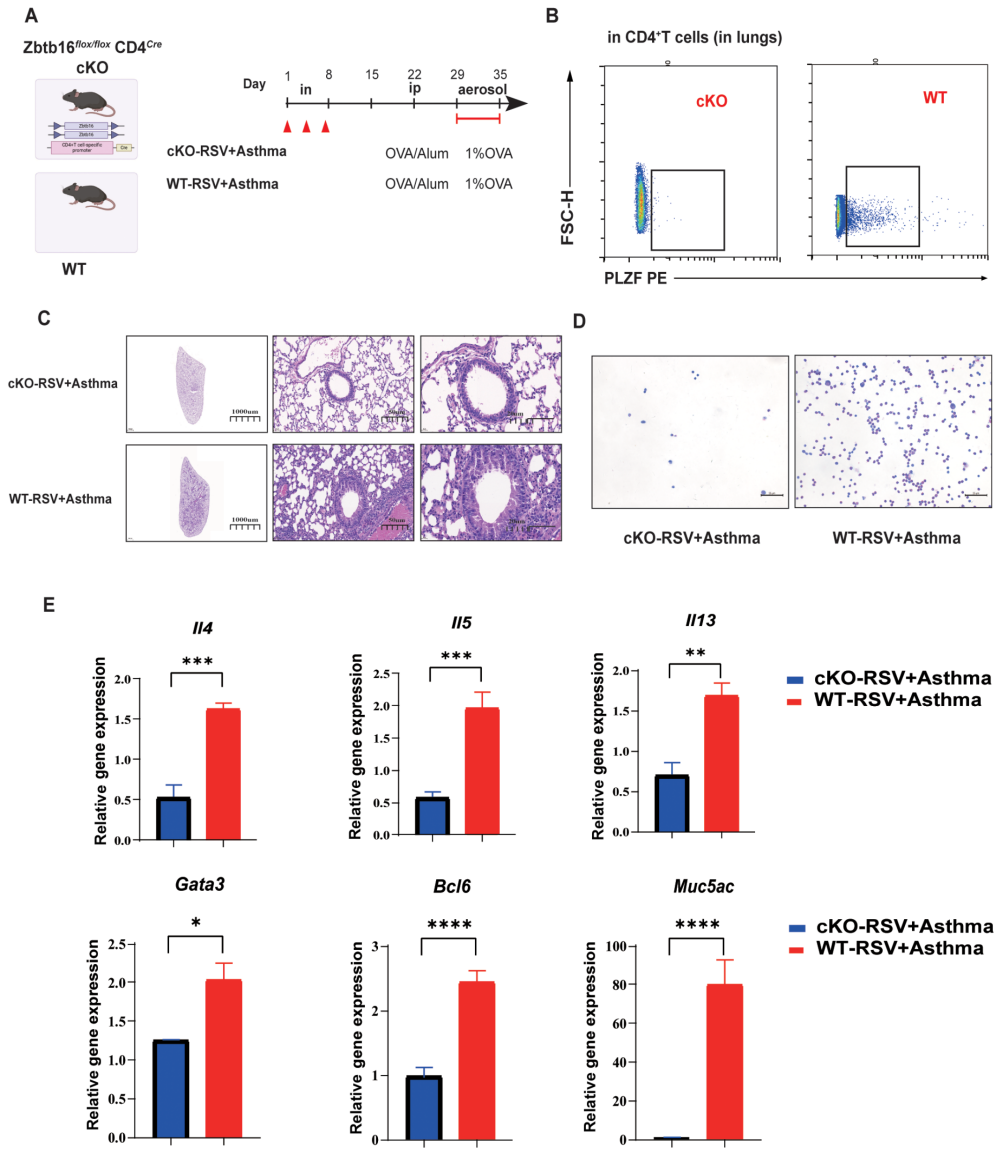
Figure 7 Conditional knockout of *Zbtb16* in CD4 ^+^ T cells in mice alleviates allergic lung inflammation aggravated by early RSV infection, accompanied by decreased Th2 responses (A) Establishment of RSV-infected asthma models in wild-type (WT) and cKO mice. (B) The knockout of Zbtb16 in CD4+ T cells in cKO mice was validated by flow cytometry. (C) Representative microphotographs of histological lung sections from cKO and WT RSV + asthmatic mice at objective magnifications of 1×, 20× and 40×. (D) BALF of cKO and WT RSV + asthmatic mice at objective magnification 10×. (E) Comparison of Th2-related cytokines and transcription factors in the lungs of WT and cKO mice. Data are shown as the mean ± SEM. *P < 0.05, **P < 0.01, ***P < 0.001, ****P < 0.0001, ns: not significant (unpaired two-tailed Student’s t test). The results are from one representative experiment of at least three independent experiments in (E) (n = 3–4 in each group).

To further elucidate the regulatory effect of PLZF on CD4 ^+^ TRMs in RSV-infected asthma, we successfully knocked out *Zbtb16* in CD4 ^+^ T cells, namely, Zbtb16 ^*flox*/ *flox*^ CD4 *^Cre^* (cKO) ( Figure 7B). RSV-infected asthma models were established in cKO and WT C57BL/6J mice ( Figure 7A). HE analysis clearly revealed swollen airways and decreased inflammatory cell infiltration ( Figure 7C,D). Fewer eosinophilic granulocytes in the cKO groups were detected in the bronchoalveolar lavage fluid ( Figure 7D). RT-qPCR revealed that the conditional knockout of *Zbtb16* in CD4 ^+^ T cells reduced the levels of Th2 cytokines such as *Il4*, *Il5*, and *Il13* and transcription factors such as *Gata3* and *Bcl6*, suggesting that PLZF could enhance allergic inflammation in the airway by increasing Th2 responses ( Figure 7E).

**Figure 8:**
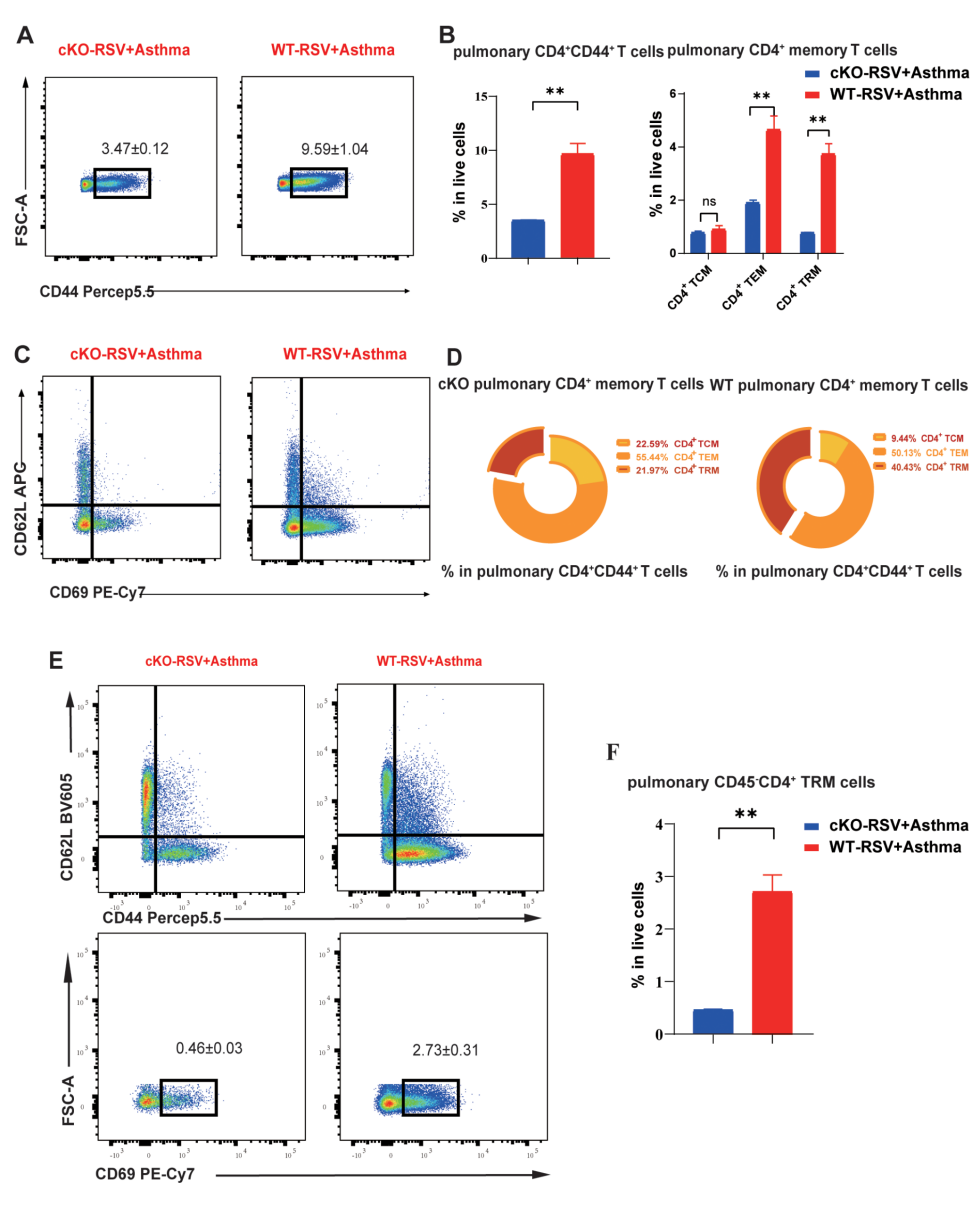
Figure 8 Comparison of pulmonary CD45 ^-^CD4 ^+^ TRM in wild-type and cKO mice (A,B) The comparison of proportion of CD4+CD44+ T cells and subtype memory T cells in live cells detected by flow cytometry. (C,D) The comparison of proportion of CD4+ TCM, CD4+ TEM and CD4+ TRMs in CD4+CD44+ T cells detected by flow cytometry. (E) General panel logic of CD45–CD4+ TRMs detected by flow cytometry. (F) The comparison of proportion of CD45–CD4+ TRMs in lungs of cKO and WT RSV + asthma mice. Data are shown as the mean ± SEM. *P < 0.05, **P < 0.01, ***P < 0.001, ****P < 0.0001, ns: not significant (unpaired two-tailed Student’s t test). The results are from one representative experiment of at least three independent experiments in (B,D,F) (n = 3–5 in each group).

Similarly, the number of pulmonary CD4 ^+^CD44 ^+^ T cells decreased in cKO mice, and among the three subtypes, CD4 ^+^ TRMs were the main contributors ( Figure 8A,B). Notably, among the three subtypes of CD4 ^+^CD44 ^+^ T cells, CD4 ^+^ TRMs were the dominant cells whose number increased in the lungs of WT mice ( Figure 8C,D). The use of CD45 better described the increased number of pulmonary CD45 ^–^CD4 ^+^ TRMs in WT RSV-infected asthmatic mice ( Figure 8E,F). CD4 ^+^ TRMs have been proven to be critical factors in the pathogenesis of asthma through the secretion of Th2 cytokines. These results indicate that PLZF can regulate CD4 ^+^ TRMs to influence Th2 responses in the pathogenesis of RSV-infected asthma.

## Discussion

Th2-high asthma is a chronic airway disease with severity ranging from occasional mild wheezing to life-threatening airway closure [30]. Asthma has high mortality if not treated promptly [31]. Early RSV infection is a critical pathogenic factor in the aggravation of asthma. At present, there are numerous available studies on the mechanisms and relationships between asthma and RSV infection. However, most studies focused on acute asthma attack and asthma exacerbation caused by RSV infection. Few studies focused on the role of memory T cells in the continuous effects of RSV infection on asthma. Moreover, the role and mechanism of RSV infection in long-term chronic inflammation in asthma remain unclear. The identification of specific targets that mediate long-term inflammation will facilitate the development of novel therapeutic approaches and improve responses to existing therapies. In this study, we determined the role of CD4 ^+^ TRMs in RSV-infected asthma and how the transcription factor PLZF regulates CD4 ^+^ TRMs and affects chronic inflammation in asthma. Our study demonstrated that the influence of RSV infection on asthma is long-lasting, with an increase in pulmonary Th2 cytokine secretion instead of B-cell-related IgE secretion. CD4 ^+^CD44 ^+^ T cells are closely related to the severity of asthma aggravated by RSV infection, and among the memory T cells, the enrichment of CD4 ^+^ TRMs in the lungs is the dominant change, which promotes the recurrence of early asthmatic pulmonary inflammation. Furthermore, overexpression of PLZF *in vitro* significantly increased the number of CD4 ^+^ TRMs, likely leading to asthma exacerbation.

RSV is a critical factor contributing to the development of asthma. Early infancy is a critical period in lung development, and severe RSV infections are associated with long-term pulmonary sequelae. Makrinioti *et al*. [30] reported that RSV bronchiolitis is likely to lead to the development of recurrent wheezing, which is closely related to asthma in their meta-analysis. Early-life RSV infection modifies the lung structure, leading to decreased lung function. Consistent with previous studies, our study revealed the continuous pathogenic role of RSV infection in asthma exacerbation lasting for at least 6 weeks. Despite long-term alleviation of symptoms, the lungs of RSV-infected asthmatic mice presented incredibly constructed and swollen airways with inflammatory cell infiltration, accompanied by high levels of Th2 cytokines such as IL-4 and IL-5. RSV-specific TRMs are traditionally regarded as safeguards against reinfection. Neonatal RSV-specific CD8 ^+^ T cells increase the expression of tissue-resident markers and are maintained in the lungs at memory time points, which is correlated with more rapid control of the virus in the lungs after reinfection [ 29, 32, 33] . Kinnear *et al*. [34] also reported that TRMs are protective against RSV reinfection. RSV reinfection symptoms can be greatly mitigated by increasing the number of RSV-specific TRMs, such as decreasing body weight and the replicated virus load. The accumulation of pulmonary virus-specific CD8 ^+^ TRMs was observed during the recovery from RSV infection [35]. Most researchers have focused on CD8 ^+^ TRMs. CD4 ^+^ TRMs are also abundant in the lungs and play a vital role. Our study revealed that asthmatic BALB/c mice infected with RSV presented more severe lung inflammation and more CD4 ^+^ TRMs than asthmatic mice did. Th2-related inflammatory cytokines, such as IL4 and IL5, are increased in asthma patients with RSV infection.

Interestingly, CD4 ^+^ TRMs are highly expressed in mice with RSV infection or asthma, whereas they act differently in RSV-infected patients and asthma patients [ 34, 36] . Independent of circulating immune memory cells, CD4 ^+^ TRMs are considered the primary reactive immune memory cells in the early stage, providing protection against RSV re-infection while contributing to asthma pathogenesis [ 17, 34] . However, whether CD4 ^+^ TRMs are protective or pathogenic in RSV-related asthma is still unknown. Our findings revealed that CD4 ^+^ TRMs were more highly expressed in the lungs of RSV-infected asthma model mice than in those of circulating CD4 ^+^ memory T cells, including CD4 ^+^ TEMs and CD4 ^+^ TCMs, for at least 6 weeks in BALB/c mice. Consistent differences were observed in the lungs of asthmatic and RSV-infected asthmatic mice. Importantly, a low concentration of antigens can stimulate asthmatic changes in the lung, such as increased inflammatory cell infiltration with many CD4 ^+^ TRMs.

PLZF, a transcription factor encoded by *Zbtb16*, plays a significant regulatory role in cell proliferation, development, and organ differentiation [34]. PLZF can regulate CD4 ^+^ memory T cells. PLZF ^+^CD4 ^+^ T cells can generate activated/memory-like regulatory T cells in lymphocytes and spleens [37]. In addition, human CD4 ^+^ T cells with PLZF expression exhibit the characteristics of terminally differentiated effector memory CD4 ^+^ T cells [38]. Our findings are similar to those of earlier studies. We demonstrated that the lungs of RSV-infected asthmatic mice express far more PLZF ^+^CD4 ^+^ TRMs than those of asthmatic mice in both the acute and memory models. *In vitro*, transient transfection with *PLZF* in primary cultured spleen cells promoted CD4 ^+^ TRMs. CD4 ^+^ TRMs contribute to recurrent asthma. It is reasonable to speculate that PLZF could promote CD4 ^+^ TRMs and aggravate asthma. Here, we utilized cKO mice to better understand the role of PLZF in RSV-infected asthmatic mice and the regulatory role of PLZF in CD4 ^+^ TRMs, which is closely related to Th2 responses in asthma. However, our previous study revealed that PLZF could promote immune tolerance through downregulating the memory phenotype and decreasing the numbers of CD4 ^+^ TEM and CD4 ^+^ TCMs [22]. Two possibilites could be proposed for these contradictory phenomena. The first possibility is the differentiation of CD4 ^+^ memory T cells with different markers. Li *et al*. [22] utilized CD44 and CD62L to distinguish CD4 ^+^ TCM and CD4 ^+^ TEMs without CD4 ^+^ TRMs. Conversely, CD44, CD62L and CD69 have been used to define CD4 ^+^ TCM, CD4 ^+^ TEM and CD4 ^+^ TRMs, which contributes to this difference. PLZF in different cells has different functions. PLZF in iNKT cells promotes immune tolerance to inhibit asthma, but PLZF restricts intestinal ILC3 function to downregulate gut immune homeostasis [21]. The second possibility is that PLZF has various effects on the proliferation, development and function of memory T cells. The amounts may not reflect the function. In our study, although the percentages of CD4 ^+^ TEMs and CD4 ^+^ TCMs were not significantly different between the PLZF-overexpressing group and the NC group, the differences in Th2 cytokine secretion in these cells should be further explored.

In summary CD4 ^+^ TRMs are vital pathogenic factors in asthma exacerbation induced by RSV infection. Overexpression of PLZF *in vitro* induces the development of CD4 ^+^ TRMs. When suitable ligands are used to selectively target the transcription factor PLZF in CD4 ^+^ TRMs, new targeted drugs may be discovered, which provides broad prospects for the clinical development of asthma treatments.
